# Therapeutics Beyond Delivery: Repurposed Drugs and Biologic Pathways

**DOI:** 10.1007/s11906-026-01392-5

**Published:** 2026-09-21

**Authors:** Michael Wilkinson, Jenny Myers

**Affiliations:** Maternal and Fetal Health Research Centre, University of Manchester, St Mary’s Hospital, Oxford Road, M13 9WL Manchester, United Kingdom

**Keywords:** Pre-eclampsia, treatment, biologic, novel, placental dysfunction

## Abstract

**Purpose of Review:**

Pre-eclampsia remains a leading cause of maternal and perinatal morbidity and mortality worldwide, yet no disease-modifying therapy currently exists. This review examines the challenges inherent in developing therapeutics for pre-eclampsia, and evaluates the evidence for repurposing existing drugs and developing novel biologic strategies to treat preterm pre-eclampsia.

**Recent Findings:**

Metformin has demonstrated a trend towards prolongation of pregnancy in a randomized controlled trial, with larger trials ongoing. Proton pump inhibitors and sulfasalazine show compelling preclinical efficacy, though neither has yet translated to clinical benefit, possibly reflecting inadequate drug exposure in vivo. Novel biologic approaches, including sFlt-1-targeting siRNA, complement inhibition with eculizumab, and placenta-tropic lipid nanoparticle delivery systems, represent promising emerging strategies.

**Summary:**

Advancing molecular classification of pre-eclampsia is essential to identifying targetable pathways and selecting patients most likely to benefit from specific interventions. Improved pharmacokinetic evaluation in pregnancy, earlier identification of at-risk women using circulating biomarkers, and regulatory frameworks supportive of obstetric research will be critical to translating promising candidates into effective therapies.

## Introduction

Pre-eclampsia is a multisystem disorder of pregnancy, characterized by widespread endothelial dysfunction resulting in hypertension and end-organ dysfunction. Complications of pre-eclampsia are life-threatening, and include neurological (eclampsia, stroke), hematological (thrombocytopenia, disseminated intravascular coagulation), renal (acute kidney injury), hepatic (hepatocellular destruction) and fetoplacental (placental abruption, fetal growth restriction, stillbirth) complications.

The current standard of care is treatment of hypertension with antihypertensive agents considered safe in pregnancy (e.g. labetalol, nifedipine), intensive monitoring of both fetal and maternal condition, and carefully timed delivery balancing the risk of preterm birth to the infant and the risk of severe complications of pre-eclampsia to the mother [1]. No disease-modifying therapy is currently available that would reduce the risk of maternal complications while allowing prolongation of pregnancy to improve perinatal outcomes.

The etiology of pre-eclampsia is heterogeneous; the syndrome of hypertension and end-organ dysfunction is dependent on the placenta and its interaction with the maternal endothelium; however, the mechanism by which endothelial dysfunction is induced is increasingly recognized to be more diverse than previously thought. Pre-eclampsia has traditionally been subdivided into early-onset (before 34 weeks gestation) and late-onset (after 34 weeks gestation). Early-onset pre-eclampsia is commonly associated with fetal growth restriction, resulting from inadequate remodeling of the maternal spiral arteries in early pregnancy and subsequent placental ischemia [2], and is associated with an imbalance of important angiogenic (placental growth factor, PlGF) and anti-angiogenic (soluble fms-like tyrosine kinase-1, sFlt-1) factors [3]. Late-onset pre-eclampsia is less well characterized, and seems to be more associated with pre-existing maternal cardiometabolic dysfunction [4], with a milder phenotype of placental stress triggering endothelial dysfunction [5].

More recently multi-omic analysis has been employed in an attempt to develop our understanding of the pathophysiology of pre-eclampsia based on its underlying molecular biology. Than et al. propose four molecular clusters corresponding to different clinical phenotypes, based on longitudinal proteomics and corresponding placental transcriptomics: metabolic, associated with early-onset disease and a pro-thrombotic state; maternal anti-fetal rejection, associated with recurrent pre-eclampsia and a failure of immune tolerance; extracellular matrix-related, associated with mild late-onset disease; and the classical placental pre-eclampsia, associated with early-onset fetal growth restriction and angiogenic imbalance [6]. Advancing our understanding of the mechanisms underlying the clinical syndrome is essential to the development of effective disease-modifying therapies, and in selecting those women most likely to benefit from a particular therapy (Table 1).

**Table 1:** Proposed molecular phenotypes of pre-eclampsia

Molecular phenotype	Affected pathways	Clinical features
Metabolic	Insulin and apelin signaling, prothrombotic	High rate of early-onset disease, SGA neonates
Maternal anti-fetal rejection	Fluid shear stress and atherosclerosis, platelet activation, proinflammatory	High BMI and high rates of recurrent pre-eclampsia, predominantly late-onset disease
Extracellular matrix-related	Focal adhesion, extracellular matrix receptor interaction	Highest rate of late-onset disease, mildest phenotype
Placental	HIF-1, VEGF, TNF, PI3K-Akt signaling	Early-onset disease, highest blood pressure, lowest birthweight

Adapted from Than et al. (2023)

## Challenges in drug Development

### Clinical Heterogeneity

The wide range of molecular pathways underlying the development of pre-eclampsia and their complex interactions remain poorly understood. We have limited biomarkers that are only useful in preterm disease, and lack additional biomarkers that could be used to identify the pathology underlying the syndrome; these would facilitate the development of targeted therapeutics and the selection of patients likely to benefit from them.

### Time Constraints

Pregnancy is by nature time constrained, and this is exaggerated in pre-eclampsia; the average time from diagnosis of preterm pre-eclampsia to delivery is around 10 days [7]. This significantly restricts the opportunity for intervention to get the disease under control, and it may be that once a diagnosis is made the disease is already too advanced to effectively restrain it. Research is ongoing into preventative tools, which must go hand-in-hand with therapeutics.

### Safety Concerns

Pregnant women are usually excluded from early-phase clinical trials due to concerns about fetal safety. Many drugs used routinely in pregnancy are used off-label, and over 80% of pregnant women receive therapeutics that have not been adequately studied in pregnancy [8]. It can be difficult to satisfy regulatory requirements for clinical trials in pregnancy, with concerns about the long-term impact of new therapeutics on the developing fetus from both regulators and sponsors complicating the approvals process. This creates a paradox where a lack of safety data in pregnancy obstructs the generation of new evidence.

Recruitment to drug trials in pregnancy can also be challenging, dependent on the participant’s perception of the balance of risk and benefit. For example, the STRIDER (Sildenafil Therapy in Dismal Prognosis Early-Onset Fetal Growth Restriction) trial completed recruitment on time and had a consent rate of 80%, where the risk of a poor outcome without treatment was extremely high, with a stillbirth rate of around one third [9]. In contrast, a recent trial of an investigational drug to treat intrahepatic cholestasis of pregnancy (ICP) invited over 1000 women to participate, with only four women receiving the drug [10]. This is likely partly due to the lower stakes in ICP, where the risks without treatment are lower and therefore the benefit of treatment perceived as smaller, as well as the difference between trialing a new molecule versus repurposing an already widely-used drug. For this reason, many clinical trials in pregnancy are now focused on repurposing existing drugs, and we will focus on those in this review.

### The Unique Human Placenta

Pre-eclampsia is mediated through or triggered by the placenta. Placental structure varies widely across mammals, with a hemochorial placenta found in humans, primates, rodents and rabbits. The deep interstitial trophoblastic invasion that is deficient in pre-eclampsia is seen only in great apes, and spontaneous pre-eclampsia is unique to humans [11]. This makes studying novel therapeutics in animal models difficult, with a limited number of rodent models available that recapitulate only some of the features of pre-eclampsia [12].

### Pharmacokinetics

From early pregnancy there are marked changes in maternal hemodynamics, including a fall in systemic vascular resistance, an increase in plasma volume of 45–50%, and a 40–50% increase in cardiac output mediated by an increase in heart rate and stroke volume [13]. A failure of the physiological expansion of cardiac output has in fact been implicated in the pathophysiology of pre-eclampsia [14]. This results in an increase in renal blood flow, with a corresponding increase in both renal size and glomerular filtration rate, which rises by 40–50% by the end of the first trimester [13]. Progesterone and estradiol, produced in high levels throughout pregnancy, induce cytochrome P450 expression [15], enhancing hepatic drug metabolism. These factors act together to reduce serum drug concentrations in pregnant women compared to non-pregnant patients, and the pharmacokinetics of any new therapy must be carefully evaluated in pregnancy to ensure therapeutic levels are achieved.

Drug concentrations in the fetoplacental compartment are altered by both changes in maternal metabolism and excretion, and by the syncytiotrophoblast which forms the fetomaternal interface. Small, lipophilic molecules will freely diffuse across the placental barrier, with variable enzymatic inactivation [16]. Some drugs are actively transported across the placenta, including monoclonal antibodies transported by the neonatal Fc receptor [17]. A growing interest in targeted biologic therapies and oligonucleotides necessitates careful evaluation of placental transfer to predict fetal effects, with ex-vivo placental perfusion [18] and increasingly sophisticated physiologically based pharmacokinetic (PBPK) modeling [19] becoming vital tools. Sildenafil failed to improve umbilical artery Dopplers or prolong pregnancy in FGR in the STRIDER trial at a dose of 25 mg three times daily [20], which is likely to be subtherapeutic – a subsequent study that evaluated paired maternal and fetal blood samples demonstrated only 3.6% of maternal sildenafil passed into the fetal circulation [21], and modeling based on animal studies suggested an optimal dose of 200–300 mg/day [22]. This again highlights the importance of thorough pharmacokinetic evaluation prior to embarking on a therapeutic trial in pregnancy, particularly where the fetus is the target of the therapy.

## Repurposing Drugs to Treat Pre-Eclampsia

Low-dose aspirin has been widely repurposed in women at risk of developing pre-eclampsia, following demonstration in the ASPRE (Aspirin for Evidence-Based Preeclampsia Prevention) trial that treatment from early pregnancy roughly halves the incidence of preterm pre-eclampsia [23]. There has been significant interest in repurposing other drugs as a prevention tool, including pravastatin [24], low-molecular-weight heparin [25], and hydroxychloroquine [26]. None has yet shown a clear efficacy signal, and we still have no effective prophylaxis for term pre-eclampsia. We will focus here on repurposing drugs in the treatment, rather than the prevention, of pre-eclampsia.

Given the goal of therapy would be to slow, stop or reverse the progression of the disease to prevent maternal complications, with the aim of prolonging pregnancy to reduce the neonatal complications of prematurity, the evidence discussed pertains to preterm pre-eclampsia. Term pre-eclampsia should be managed with prompt delivery [1], and while prevention is important it presents less of a therapeutic challenge.

### Metformin

Metformin currently has the most developed evidence supporting its use as a therapy for preterm pre-eclampsia. As discussed above, at least a subset of patients with pre-eclampsia have significant metabolic dysfunction involving insulin and apelin signaling, and this is associated with early-onset disease [6]. Metformin may ameliorate this metabolic dysfunction, and has been shown to suppress hypoxia inducible factor-1 production [27] by downregulating mitochondrial electron transport chain activity [28]. This may explain its effect on placental tissue in vitro, where it seems to reduce production of the circulating anti-angiogenic factors sFlt-1 and soluble endoglin (sEng) implicated in pre-eclampsia [29].

A double-blind, randomized controlled trial of extended-release metformin vs. placebo to treat preterm pre-eclampsia demonstrated a trend towards a prolongation in pregnancy, with a median difference of 7.6 days, although this did not reach statistical significance [7]. This study was not powered to detect differences in maternal or perinatal outcomes, although there was a reduction in length of neonatal stay in the metformin arm, and did not reproduce the reduction in circulating levels of sFlt-1 and sEng in vivo that was seen in vitro. A larger trial is underway in the same setting in South Africa [30], alongside a European trial in Sweden (NCT06033131).

### Proton pump Inhibitors

Proton pump inhibitors (PPIs) have also been investigated as a therapy in pre-eclampsia. Omeprazole is widely used in pregnancy for acid reflux, with a good safety profile [31]. There is convincing evidence in a range of preclinical models that PPIs, and esomeprazole in particular, constitute a promising therapeutic candidate; they reduce sFlt-1 and sEng production and reduce inflammation in placental tissue, seem to improve endothelial function, dilate maternal blood vessels in vitro and decrease blood pressure in a mouse model of pre-eclampsia [32]. The exact mechanism by which these effects are mediated remains elusive; however, there is certainly a downregulation of sFlt-1 at a transcriptional level, and evidence that suppression of the renin angiotensin system in the placenta through inhibition of the endocytic receptor megalin (responsible for renin and angiotensinogen uptake) is important [33].

Observational studies attempting to ascribe a change in the incidence of pre-eclampsia to administration of PPIs taken for other indications have variously reported an increase, decrease, and no difference in incidence [34, 35].

Unfortunately, the promising results from preclinical studies have not been borne out in clinical trials. A randomized controlled trial of esomeprazole in women with preterm pre-eclampsia failed to demonstrate a prolongation of pregnancy compared to placebo, and while placental abruptions were more frequent in the placebo group this was not significant in an adjusted analysis [36]. Circulating levels of sFlt-1 were not suppressed in vivo, and this was replicated in a trial of omeprazole which showed no alterations in circulating sFlt-1, PlGF, or endothelin-1 (ET-1), although the duration of treatment was only four days [37].

There may still be hope for PPIs – serum levels of esomeprazole only reached the lower range of concentrations that were effective in vitro in the PIE trial [36], and a higher dose may be more effective, underlining the importance of careful pharmacokinetic evaluation in pregnancy. Alterations in circulating sFlt-1 and PlGF levels have been shown to precede the development of the clinical syndrome of pre-eclampsia by around 5 weeks [3]. Increasing use of circulating factors in the diagnosis [38] and screening (ISRCTN71774663) of pre-eclampsia may identify women earlier in the disease process, when it may be more amenable to rescue.

### Sulfasalazine

Pre-eclampsia is fundamentally an inflammatory condition, with immune dysregulation implicated in both the development and progression of the disease [39]. A failure of immune tolerance in early pregnancy partly mediated by uterine natural killer (NK) cells likely leads to the improper placentation seen in early-onset disease [40]. Chronic inflammation triggered by oxidative stress and placental ischemia-reperfusion injury results in the release of a wide range of pro-inflammatory cytokines that are consistently elevated in pre-eclampsia compared to healthy pregnancy [41].

It follows that anti-inflammatory drugs may modify the disease process. Sulfasalazine is an anti-inflammatory drug used in the treatment of inflammatory bowel disease in pregnancy, with no evidence of adverse maternal or fetal effects [42]. Its pleiotropic immunomodulatory effects are mediated by the parent molecule and its breakdown products, sulfapyridine and 5-aminosalicylic acid (5-ASA). It reduces T-cell proliferation and the production of pro-inflammatory cytokines, suppresses immunoglobulin production, and inhibits NK cell mediated cytotoxicity [43].

Proof-of-concept in vitro work has demonstrated that sulfasalazine reduces sFlt-1 and sEng secretion and increases PlGF secretion from human placental tissue, potently relaxes systemic maternal arteries, and rescues angiogenesis in mouse aortic explants treated with sFlt-1 [44]. These results should be interpreted with caution, as a recent pharmacokinetic study of sulfasalazine in women with preterm pre-eclampsia showed antiangiogenic markers continued to rise in maternal plasma despite treatment [45]. It should be noted that serum concentrations after 2–3 g daily dosing in this study were approximately ten-fold lower than the lowest effective concentration in vitro [44], however it has been previously noted that serum sulfasalazine concentrations do not correlate well with its clinical effect [43], and drug levels in a different compartment may be more relevant. Sulfapyridine appeared to preferentially accumulate in placental tissue [45], and this may still exert a disease-modifying effect.

## Biologic Treatment Strategies

An increasingly varied catalog of biologic therapies is being used to treat inflammatory conditions in pregnancy, with a growing body of evidence suggesting they are safe for mother and fetus [46]. Beyond monoclonal antibodies and small molecule inhibitors, there is growing interest in increasingly sophisticated biologics, including stem-cell therapy [47], gene therapy [48], T-cell immunotherapy [49], and targeted drug delivery systems [50], particularly in oncology. Here we will review the most promising novel strategies for the treatment of pre-eclampsia.

### sFlt-1 siRNA

Overexpression of sFlt-1 in the placenta has been linked to the development of pre-eclampsia for over 20 years [3]. It is a splice variant of the vascular endothelial growth factor receptor-1 (VEGFR1), retaining the extracellular domain that binds VEGF and PlGF with a high affinity, but lacking the transmembrane and intracellular domains rendering it soluble [51]. It therefore acts as a decoy receptor, antagonizing the angiogenic and pro-endothelial effects of VEGF, and contributing to the syndrome of pre-eclampsia.

sFlt-1 is highly expressed in the syncytiotrophoblast in normal pregnancy, and a rise in plasma levels is seen in healthy pregnancy at term. Significantly higher levels are found in the plasma of women with pre-eclampsia, and a ratio of serum sFlt-1:PlGF has been introduced clinically to aid the diagnosis of pre-eclampsia [52]. sFlt-1 clearly plays a role in the homeostasis of angiogenesis, and anti-VEGF biologic treatments mimicking the effect of sFlt-1 have revolutionized the management of proliferative retinopathies [53] and a variety of solid tumors [54]. Angiogenesis is perhaps most recognisable in the eye, and a clue to the role of sFlt-1 is seen in manatees, the only mammal with a uniformly vascularized cornea and which does not express sFlt-1 [55].

Suppressing the pathological overproduction of sFlt-1 in the placenta is therefore an attractive strategy for the treatment of pre-eclampsia, but without causing uncontrolled angiogenesis in other tissues where sFlt-1 plays an important homeostatic role. Monoclonal antibodies directed at sFlt-1 would also bind to the identical extracellular domain of VEGFR1, inhibiting its activation and exacerbating the disease. The use of small interfering RNAs (siRNA) has garnered interest given their simplicity (as compared to monoclonal antibodies and small molecule inhibitors) and exquisite target selectivity, as well as their transient, reversible effects [56].

Turanov et al. developed a siRNA specific to sFlt-1 mRNA, that avoids counterproductive silencing of VEGFR1 production. It targets two isoforms of sFlt-1 mRNA, sFlt-1-i13 (conserved across species and expressed in a variety of human tissues) and sFlt-1-e15a (specific to primates and expressed more highly in placenta) and can be delivered via subcutaneous injection. In a baboon model of pre-eclampsia, it potently reduced sFlt-1 production, as well as blood pressure and proteinuria in treated animals [57, 58].

A phase Ia trial of the sFlt-1 siRNA CBP-4888 in healthy non-pregnant women has completed recruitment (NCT05881993), and a phase Ib dose-finding study in women with preterm pre-eclampsia is currently recruiting in Australia (NCT07282171) and is soon to open in the UK. CBP-4888 has been granted fast track designation by the US FDA, and orphan drug designation by the European Medicines Agency in recognition that preterm pre-eclampsia is a rare and underserved condition; this should remove some of the regulatory stumbling blocks discussed above that have limited obstetric research in the past. siRNAs can be manufactured relatively simply and can be stored at room temperature [57], thus could provide a viable therapeutic option in low-income settings where the burden of pre-eclampsia and its complications is highest [59].

### Eculizumab

There has been recent recognition of terminal complement activation as an important factor in the development of severe pre-eclampsia. The complement system, traditionally seen as a blunt instrument in the armory of the innate immune system to destroy invading pathogens, is tightly regulated and plays an important role in homeostasis, adaptive immunity, neurodevelopment and coagulation [60]. The complement fragments C5a and C5b-9 have been found to be elevated in the urine and serum of women with severe pre-eclampsia, implicating dysregulation of the terminal complement pathway in the development of severe disease [61, 62].

The most severe end of the spectrum of pre-eclampsia is hemolysis, elevated liver enzymes and low platelets (HELLP) syndrome. This is difficult to distinguish clinically from atypical hemolytic uremic syndrome (aHUS), an extremely rare condition characterized by a thrombotic microangiopathy resulting in hemolysis, thrombocytopenia and acute kidney failure, which can be triggered by pregnancy [63]. aHUS results from unregulated complement activation, and the mainstay of modern treatment is with the complement C5 inhibitor eculizumab [64]. Given HELLP syndrome and aHUS may share complement dysregulation as an underlying mechanism, eculizumab has been proposed as a therapeutic option to arrest complement activation in severe pre-eclampsia.

A recent single-arm trial of eculizumab to treat HELLP syndrome at < 30 weeks gestation recruited three women, all of whom were delivered for fetal indications within 59 h of starting treatment, reflecting the severity of placental dysfunction. In two cases, transaminases and platelet counts started improving following eculizumab administration and prior to birth, but in the absence of a control group it is difficult to ascribe this to treatment [65]. This limited evidence suggests there may be a benefit of complement inhibition in ameliorating the maternal syndrome; however, the prospect of prolonging pregnancy in the context of such advanced uteroplacental insufficiency seems ambitious. A phase II trial of eculizumab (NCT04725812) for severe pre-eclampsia < 30 weeks was registered in 2021, and terminated in 2023. The measurement of urinary C5 fragments may present a method of identifying women at highest risk of developing severe pre-eclampsia, and those that might benefit most from anti-complement therapies.

### Placenta-Targeted Therapies

The desire to reduce the systemic toxicity associated with anti-cancer drugs has led to significant advances in the development of targeted drug delivery systems. A particularly successful strategy is the encapsulation of small molecules in a phospholipid bilayer (liposomes). Liposomal delivery of, for example, amphotericin B and doxorubicin increases delivery to the target tissue while reducing associated toxicity, and half a century of research has been dedicated to reducing immunogenicity of these preparations and increasing the selectivity of their delivery [66].

The delivery of nucleic acids is very inefficient in a liposomal system, as they are susceptible to degradation by nucleases in the aqueous core of the liposome [67]. Lipid nanoparticles (LNPs) are much more effective, lacking the aqueous core, and were used very successfully in the delivery of the COVID-19 mRNA vaccines [68]. LNPs offer a potential solution for targeted delivery of mRNA or siRNA to the placenta, minimizing off-target effects.

A recent breakthrough used high-throughput screening of LNPs to identify a placenta-tropic LNP capable of selectively delivering mRNA to trophoblast [69]. LNPs have traditionally been highly taken up in the liver, and therefore therapies have been focused on hepatic diseases, such as the FDA-approved treatment Onpattro [70]. The discovery that LNP-55 delivers mRNA in high concentrations to the placenta and spleen, and to a lesser extent the liver, may pave the way for targeted therapies in pre-eclampsia. Swingle et al. demonstrated that a single injection of LNP-55 delivering VEGF mRNA resolved maternal hypertension until the end of gestation in two different mouse models of pre-eclampsia [69]. These promising preclinical data suggest a potentially curative role of placenta-targeted nucleic acid therapy in pre-eclampsia, although the potential adverse effects of increased angiogenesis in the spleen and systemic circulation remain to be elucidated. This also offers a potential advantage over an adenoviral delivery mechanism for VEGF mRNA proposed in the treatment of FGR, which requires ultrasound-guided injection into the uterine artery rather than intravenous administration [71]. LNPs are unstable at room temperature, and are stored frozen at -80 °C, which may limit their translation in low-income settings where the clinical need for a pre-eclampsia therapy is highest. Lyophilization (freeze-drying) seems to extend their shelf life and may offer a solution for delivery in low-income countries [72].

### Bradykinin Upregulation

Bradykinin is an important stimulator of endothelium-mediated vasorelaxation in the systemic circulation. It acts on endothelial cells to increase production of vasodilators such as nitric oxide and prostacyclin, which then act on vascular smooth muscle cells to induce relaxation [73]. The effect of bradykinin is significantly blunted in pre-eclampsia, and its receptor heterodimerizes with the angiotensin-II receptor to potentiate vasoconstriction [74].

A novel strategy to upregulate bradykinin signaling is by administering DM199, a recombinant tissue kallikrein-1 analogue which is more stable than its parent molecule [75]. Kallikrein is an endogenous enzyme that cleaves kininogen into bradykinin, and thus DM199 should increase bradykinin production in the endothelium. This may also help to circumvent the sFlt-1 mediated blockade of VEGF signaling, as kallikrein has also been shown to increase VEGF and VEGFR2 production in rat myocardium [76].

**Fig. 1:**
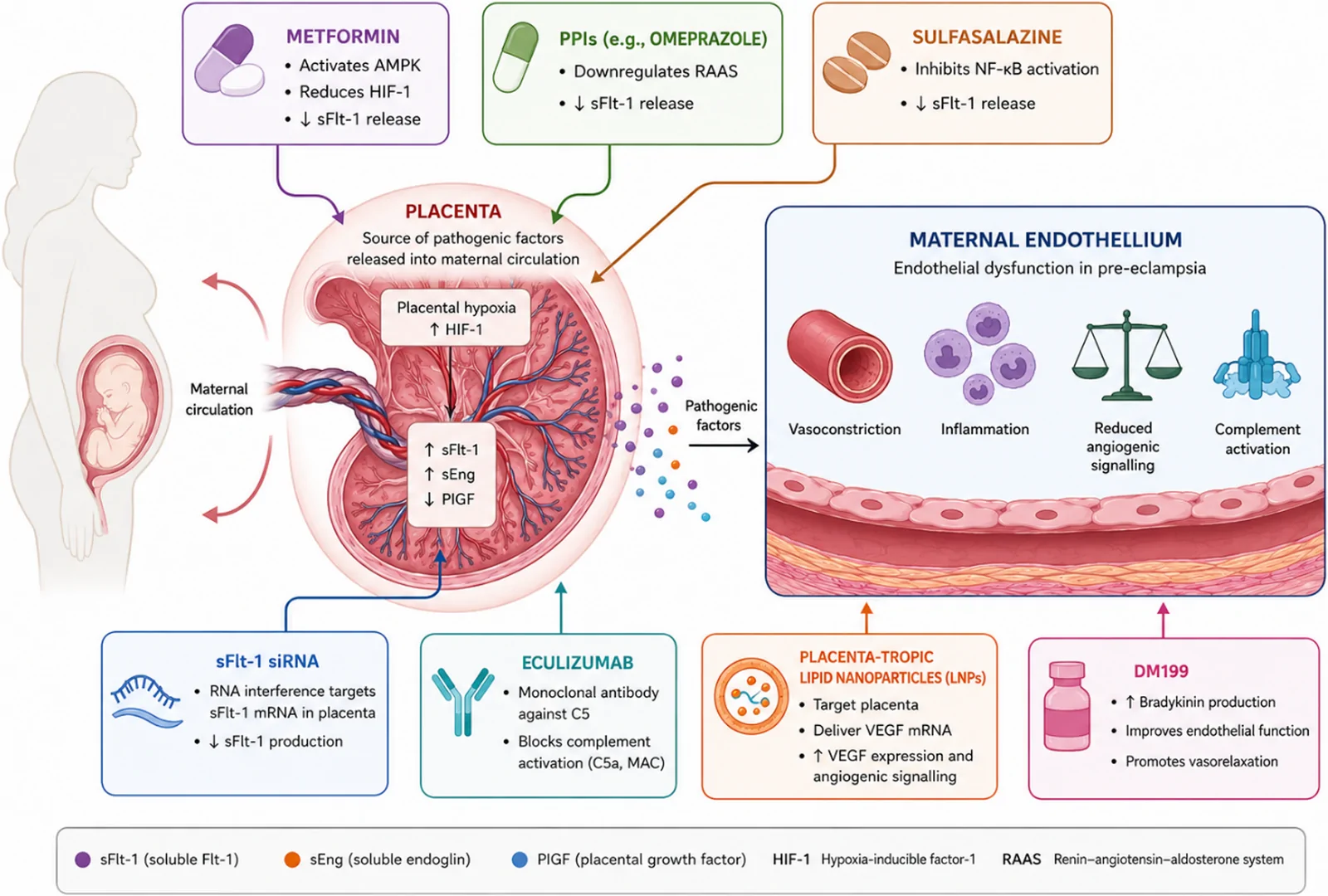
Proposed novel therapeutics for preterm pre-eclampsia

A trial is currently underway to establish a safe dose that will lower maternal blood pressure, and to measure DM199 concentrations in cord blood [75]. If DM199 does cross the placenta in significant quantities, though unlikely, it may be harmful to the fetus; higher concentrations of bradykinin counterintuitively cause vasoconstriction in the placental circulation [77], which may worsen Dopplers in fetal growth restriction. Bradykinin is a key mediator in the neonatal transition [78], and high concentrations may also lead to premature closure of the ductus arteriosus (Fig. 1) (Table 2).

**Table 2:** Proposed novel therapeutics for preterm pre-eclampsia

Proposed treatment	Mechanism of action	Preclinical data	Clinical validation
Metformin	↓ hypoxia by modulating metabolic activity	↓ sFlt-1, sEng	Trend towards prolongation of pregnancy
Proton Pump Inhibitors	↓ sFlt-1 transcription, RAAS activity	↓ sFlt-1, sEng, inflammation, increased vasodilation	No effect in phase II trial
Sulfasalazine	Anti-inflammatory	↓ sFlt-1, sEng, increased PlGF and vasodilation	No effect in phase Ib trial, more evidence needed
sFlt-1 siRNA	Silencing of sFlt-1 production	↓ sFlt-1, blood pressure and proteinuria in a baboon model	Phase Ib trial in progress
Eculizumab	Terminal complement inhibition	Limited	Trials limited by recruitment issues
LNP-55 VEGF mRNA	↑ placental VEGF production	Resolved maternal hypertension in mouse models	None
DM199	↑ bradykinin production	Limited	Phase Ib/II trial in progress

## Conclusions

Pre-eclampsia represents one of the most significant unmet needs in obstetrics. Despite considerable advances in our understanding of its molecular underpinnings, translating this knowledge into effective disease-modifying therapy has proven challenging. The heterogeneity of the syndrome, the unique pharmacokinetic environment of pregnancy, and the regulatory and ethical barriers to obstetric drug development have collectively impeded progress. 

Nevertheless, there are reasons for cautious optimism. The emerging molecular classification of pre-eclampsia offers a framework for stratified trial design, moving beyond the one-size-fits-all approach that may have obscured efficacy signals in earlier studies. Metformin remains the most clinically advanced candidate, with adequately powered trials ongoing. The development of sFlt-1-targeting siRNA, supported by fast track and orphan drug designations, represents a significant step forward for biologic approaches, while placenta-tropic lipid nanoparticle delivery systems offer the tantalizing prospect of targeted, potentially curative therapy.

Ultimately, earlier identification of women with evolving pre-eclampsia using circulating biomarkers, combined with therapies precisely matched to the underlying molecular pathology, may finally offer the opportunity to interrupt the disease process rather than simply manage its consequences.

## Perspectives and Future Directions

The field of pre-eclampsia therapeutics is at a pivotal moment. A greater understanding of the molecular heterogeneity underlying the clinical syndrome, combined with advances in targeted drug delivery and biomarker development, offers a more credible route to disease-modifying therapy than has previously existed.

Placenta-targeted drug delivery is perhaps the most promising avenue for future development. The identification of placenta-tropic LNPs capable of selectively delivering nucleic acid therapies to trophoblast offers the possibility of correcting placental dysfunction at its source while minimizing off-target effects. As the technology matures, LNPs may serve as a platform for delivering a range of therapeutic payloads — mRNA, siRNA, or small molecules — directed at the specific molecular pathology driving disease in an individual patient. The challenge of cold-chain storage in low-income settings, where the burden of pre-eclampsia is greatest, will need to be addressed if these advances are to benefit the women who need them most.

Central to future therapeutic development is a move towards personalized medicine. The molecular classification of pre-eclampsia into distinct phenotypes reframes the challenge considerably; each subtype may have differing therapeutic targets and differing likelihood of responding to a particular intervention. Identifying and validating circulating or urine biomarkers that reliably distinguish these phenotypes early enough to permit therapeutic intervention is therefore a research priority. This would enable selection of women most likely to benefit from a given therapy, and facilitate enriched recruitment to clinical trials — an important consideration given the considerable barriers to drug development in pregnancy outlined above.

## Key References

- RCT suggesting metformin may prolong pregnancy in pre-eclampsia - Cluver CA, Hiscock R, Decloedt EH, Hall DR, Schell S, Mol BW, et al. Use of metformin to prolong gestation in preterm pre-eclampsia: randomised, double blind, placebo controlled trial. BMJ. 2021;374:n2103. https://doi.org/10.1136/bmj.n2103
- Preclinical data demonstrating the efficacy of sFlt-1 siRNA in the treatment of pre-eclampsia - Turanov AA, Lo A, Hassler MR, Makris A, Ashar-Patel A, Alterman JF, et al. RNAi modulation of placental sFLT1 for the treatment of preeclampsia. Nat Biotechnol. 2018;36:1164–73. https://doi.org/10.1038/nbt.4297
- Preclinical data suggesting the potentially curative role of VEGF mRNA lipid nanoparticles in pre-eclampsia - Swingle KL, Hamilton AG, Safford HC, Geisler HC, Thatte AS, Palanki R, et al. Placenta-tropic VEGF mRNA lipid nanoparticles ameliorate murine pre-eclampsia. Nature. 2025;637:412–21. https://doi.org/10.1038/s41586-024-08291-2

## Data Availability

No datasets were generated or analysed during the current study.
